# Lipid Transport in Brown Adipocyte Thermogenesis

**DOI:** 10.3389/fphys.2021.787535

**Published:** 2021-12-23

**Authors:** Gina Wade, Ayren McGahee, James M. Ntambi, Judith Simcox

**Affiliations:** Department of Biochemistry, University of Wisconsin-Madison, Madison, WI, United States

**Keywords:** brown adipose tissue (BAT), fatty acid, fatty acid binding protein (FABP), triglycerides (TGs), CD36, thermogenesis, lipoprotein, fatty acid transport protein (FATP)

## Abstract

Non-shivering thermogenesis is an energy demanding process that primarily occurs in brown and beige adipose tissue. Beyond regulating body temperature, these thermogenic adipocytes regulate systemic glucose and lipid homeostasis. Historically, research on thermogenic adipocytes has focused on glycolytic metabolism due to the discovery of active brown adipose tissue in adult humans through glucose uptake imaging. The importance of lipids in non-shivering thermogenesis has more recently been appreciated. Uptake of circulating lipids into thermogenic adipocytes is necessary for body temperature regulation and whole-body lipid homeostasis. A wide array of circulating lipids contribute to thermogenic potential including free fatty acids, triglycerides, and acylcarnitines. This review will summarize the mechanisms and regulation of lipid uptake into brown adipose tissue including protein-mediated uptake, lipoprotein lipase activity, endocytosis, vesicle packaging, and lipid chaperones. We will also address existing gaps in knowledge for cold induced lipid uptake into thermogenic adipose tissue.

## Introduction

Endotherms maintain their body temperature by producing heat through both shivering and non-shivering thermogenesis. The cells primarily involved in non-shivering thermogenesis include brown and beige adipocytes, both of which stimulate heat production through disruption of the mitochondrial proton gradient by uncoupling protein 1 (UCP1) (Cannon and Nedergaard, 2004; Cannon et al., 2020). These cells leverage other mechanisms to produce heat including futile cycling of phosphocreatine, calcium, and free fatty acids (Prentki and Madiraju, 2008; Kazak et al., 2015; Ikeda et al., 2017, 2018). The high energy demand of these futile cycles makes cells reliant on peripheral sources of stored fuels including glucose and lipids.

Research on peripheral fuel sources for non-shivering thermogenesis has focused on glucose uptake by brown and beige adipose tissue. Until the late 1980s it was thought that only human infants contained significant quantities of brown adipose tissue (BAT) (Lean et al., 1986). However, innovation in imaging with positron emission tomography with contrast tomography (PET-CT) using ^18^F-fluorodeoxyglucose demonstrated that adult humans also have BAT capable of cold-induced heat production (active BAT) (Saito et al., 2009; van Marken Lichtenbelt et al., 2009; Virtanen et al., 2009; Von Bank et al., 2021a). The use of glucose uptake to image and quantify BAT mass and function has led to a glucose centric view of thermogenesis. This has been fortified by the discovery that BAT is able to regulate whole body glucose homeostasis in humans and takes up 8-fold more glucose than skeletal muscle when activated (Chondronikola et al., 2014; Sidossis and Kajimura, 2015; Carpentier et al., 2018). Moreover, adult humans that are exposed to repeated cold exposure have significantly lower blood glucose and hemoglobin A1C levels, the effects of which are dependent on the presence and quantity of BAT (Matsushita et al., 2014; Hanssen et al., 2016). Activation of thermogenesis with β3-adrenergic receptor agonists showed a similar response, leading to improved glucose levels in obese and diabetic individuals (Cypess et al., 2015; Finlin et al., 2020). Recent studies in humans and mice demonstrate that cold activation of BAT leads to import of fuel sources other than glucose including non-esterified fatty acids, branch chain amino acids, and triglycerides (TGs) (Bartelt et al., 2011; Ouellet et al., 2012; Yoneshiro et al., 2019).

**Figure 1:**
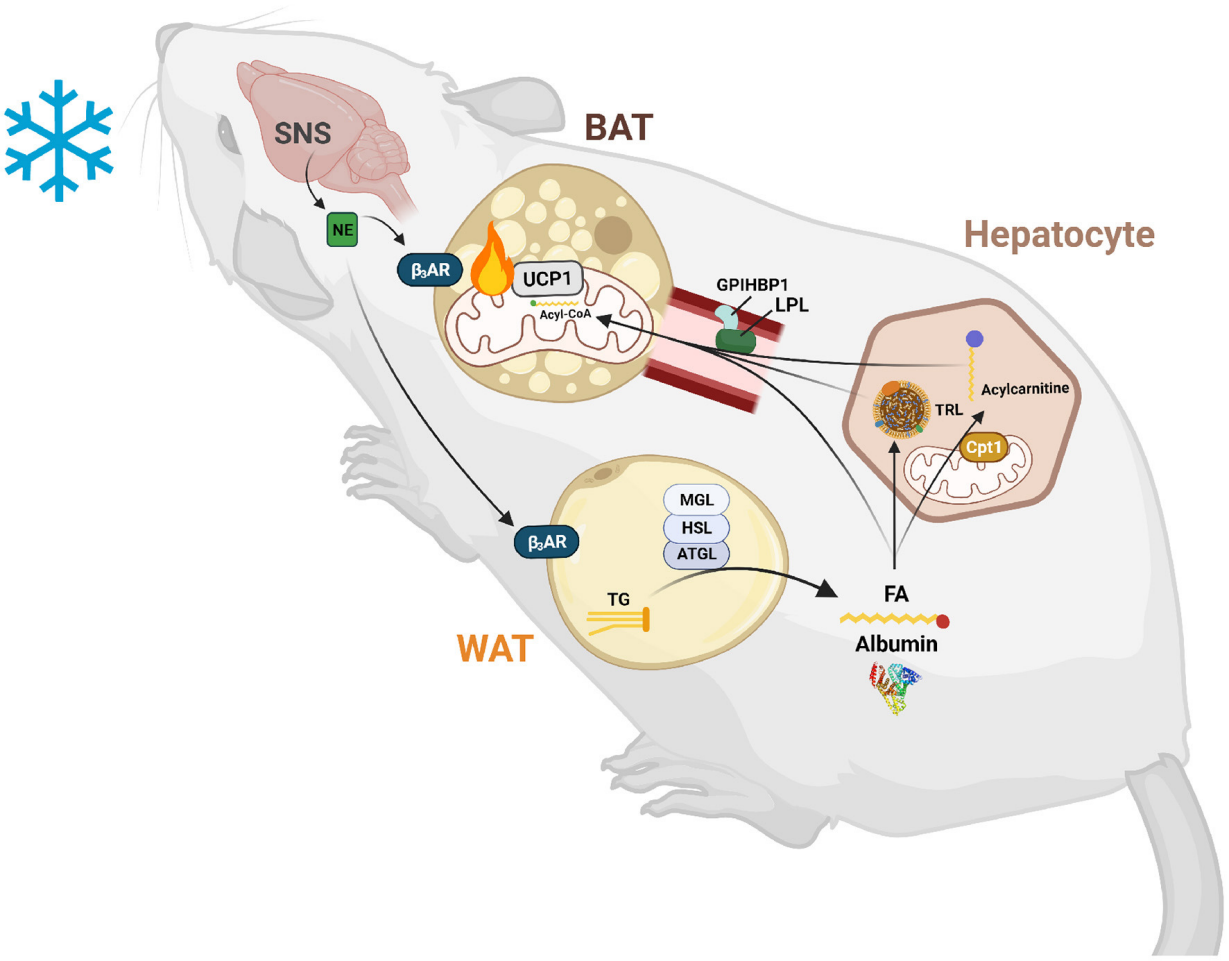
Lipid-mediated crosstalk between the liver, WAT, and BAT is required for non-shivering thermogenesis. After exposure to cold temperatures, the sympathetic nervous system (SNS) releases norepinephrine (NE) to signal a need for thermogenesis. NE binds to β3-adrenergic receptors (β_3_ARs) in white and brown adipose tissue (WAT and BAT). In WAT, this signal induces lipolysis of triglycerides (TGs) into fatty acids (FAs) and glycerol by an intracellular neutral lipase cascade involving adipose triglyceride lipase (ATGL), hormone sensitive lipase (HSL), and monoglyceride lipase (MGL). These FAs are exported into the circulation and are either taken up by BAT to activate uncoupling protein 1 (UCP1) and be broken down by β-oxidation or are taken up by hepatocytes to serve as substrates and transcriptionally activate acylcarnitine production. These FAs are also used to synthesize TGs for packaging into triglyceride-rich lipoproteins (TRLs) for export into the circulation and use by BAT to fuel thermogenesis. Hepatic acylcarnitines synthesized after signaling by WAT through FAs are exported into the circulation to be used as both a signal and fuel for heat production.

Circulating lipids are also required as a fuel source for BAT (Figure 1). The most common circulating lipids are triglycerides, cholesterol and cholesteryl esters, and phospholipids. These lipids make up lipoproteins that are assembled by the intestine (chylomicrons and HDL) or liver (LDL, IDL, and HDL). Lipoprotein transport and uptake will be discussed further in section Extracellular Lipolysis and LDL Receptor Endocytosis Facilitates Triglyceride and Cholesterol Uptake into BAT. Free fatty acids (FFAs) also circulate at high levels bound to albumin. FFAs are released from white adipose tissue (WAT) after induction of an intracellular lipolysis cascade and taken up by peripheral tissues either passively (diffusion) or actively (protein-mediated). TG-rich lipoproteins (TRLs) such as VLDL, are the main source of circulating FFAs for BAT during thermogenesis (Festuccia et al., 2011; Hoeke et al., 2016). However, FFAs from WAT are necessary for body temperature regulation in mice (Schreiber et al., 2017). These FFAs can be directly imported into BAT or taken up by the liver where they are processed into TGs or acylcarnitines that are then shuttled to BAT (Górski et al., 1988; Simcox et al., 2017; Grefhorst et al., 2018). Moreover, hepatic uptake of FFA from WAT is necessary for activation of the transcription factor HNF4α, which positively regulates expression of genes involved in acylcarnitine synthesis. This suggests that circulating lipids are also essential signaling molecules for thermogenic activation, and lipid import into liver and BAT is required for the thermogenesis. The liver exports lipoproteins, cholesterol, and acylcarnitines into the circulation following import of FFA in response to cold exposure which are preferentially taken up by brown adipocytes (Bartelt et al., 2011; Berbée et al., 2015; Hoeke et al., 2016; Simcox et al., 2017). Collectively these studies show that a variety of lipids are taken up by BAT, which is reflected in the changing composition of circulating lipids upon cold exposure (Simcox et al., 2017; Lynes et al., 2018). This review will focus on the emerging knowledge that circulating lipids are an important mediator of thermogenic potential with a focus on how their transport into brown adipocytes is facilitated.

## Circulating Lipids are Necessary For Bat Thermogenesis

The utilization of circulating lipids by cold-activated BAT was not appreciated until 2011, when Bartelt et al. demonstrated a shuttling of TRLs into activated murine BAT (Bartelt et al., 2011). This uptake of TRLs was found to be necessary for thermogenesis. Mice unable to transport FFAs and TRLs generated by whole body knockout (KO) of the putative FA transport protein cluster of differentiation 36 (*Cd*36^−/−^) were cold intolerant. The reliance of BAT thermogenesis on circulating lipids was further demonstrated by genetic ablation of the first enzyme in intracellular TG lipolysis, adipose triglyceride lipase (ATGL) encoded by *Pnpla2*. Whole body or adipose tissue specific ATGL KO led to accumulation of TGs in BAT and an inability to maintain body temperature (Haemmerle et al., 2006). Conversely, loss of ATGL in brown and beige adipocytes alone had no effect on body temperature or thermogenic transcripts (Schreiber et al., 2017). The results from the ATGL KO mouse models tell a compelling story that lipolysis in brown and beige adipocytes is dispensable for body temperature maintenance, but lipolysis in WAT is required. These findings were supported in mice unable to synthesize TGs and subsequently lipid droplets in BAT due to a UCP1-cre driven knockout of acyl-CoA:diacylglycerol transferase (*Dgat*) 1 and 2 (Chitraju et al., 2020). In this model, body temperature was maintained in the cold and utilization of circulating FFAs, and glucose was increased in BAT. Together, these studies supported the need for lipid uptake from plasma into BAT for thermogenic regulation.

Just as BAT uptake of glucose regulates systemic glucose homeostasis, uptake of lipids into BAT also regulates the circulating lipid pool. In hyperlipidemic mice modeled by KO of the extracellular lipolysis stimulator apolipoprotein A-V (*Apoa5*), cold exposure led to reduced levels of TGs and cholesterol in the plasma accompanied by an influx into the BAT (Bartelt et al., 2011; Berbée et al., 2015). These data demonstrate that BAT is an important regulator of the circulating lipid pool in mice (Hoeke et al., 2016). The presence of BAT in humans also regulates circulating TG and high-density lipoprotein (HDL) cholesterol levels. Moreover, repeated cold exposure has been shown to normalize circulating lipid levels and decrease hepatic lipid accumulation in humans (Wang et al., 2015; Bartelt et al., 2017; Wibmer et al., 2021). Although there is overwhelming evidence that cold activated BAT relies on lipid uptake for thermogenesis and can modulate circulating lipid levels, little is known about the regulation of transporters and the mechanisms that control lipid uptake. The next sections will focus on the protein-mediated uptake of FFAs, TGs, and other circulating lipids into activated BAT.

## Free Fatty Acid Uptake Into Bat is Mediated by Fabp, Fatp, and CD36

**Figure 2:**
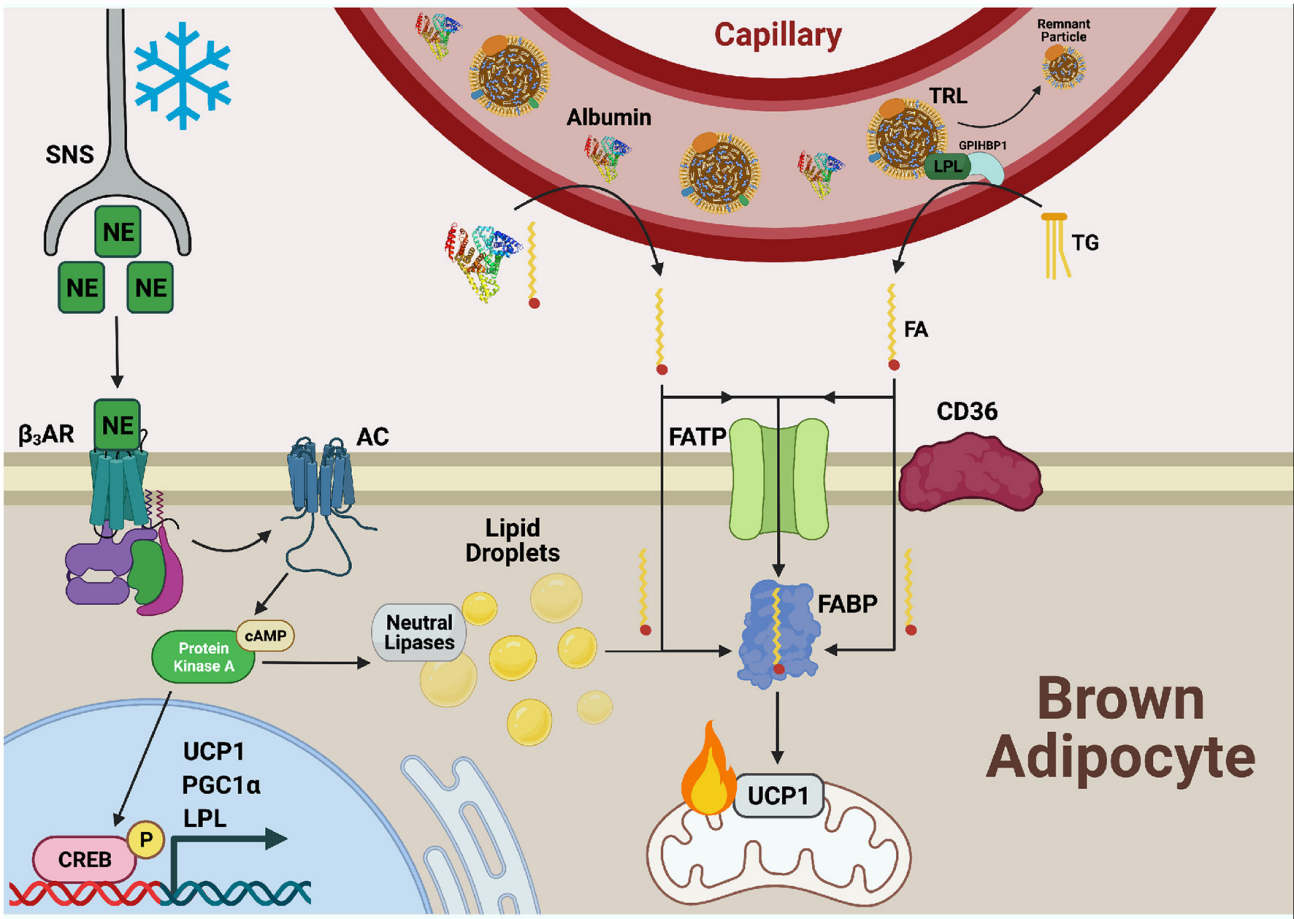
Fatty acid uptake is facilitated by FATP, CD36, and FABP_PM_ in cold-activated BAT. Cold exposure stimulates the release of norepinephrine (NE) from the sympathetic nervous system (SNS) which is sensed by β3-adrenergic receptors (β_3_ARs) on the cell surface of adipocytes. Adenylate cyclase (AC) is activated and produces cyclic AMP (cAMP) for binding to protein kinase A (PKA). This upregulates transcriptional programs to support thermogenesis, including uncoupling protein 1 (UCP1), proliferator-activated receptor-gamma co-activator (PGC1α), and lipoprotein lipase (LPL) through cAMP-responsive element binding protein (CREB). PKA also activates lipolysis of lipid droplets to liberate fatty acid (FAs), fuel FA β-oxidation, and activate UCP1 through direct binding. Necessary to this process is the transport of exogenous FAs into BAT by the combined action of fatty acid transport proteins (FATPs), CD36, and fatty acid binding proteins (FABPs). FAs bound to albumin are released from the circulation into the brown adipocyte and can either diffuse across the plasma membrane or be actively transported by the FA transport machinery. Triglycerides (TGs) from circulating TG rich lipoproteins (TRLs) are hydrolyzed to FAs via LPL tethered to the vascular lumen by glycophosphatidylinositol-anchored HDL-binding protein 1 (GPIHBP1) for transport into and use by the cell as well.

FA transport is divided into 3 steps: (1) adsorption, (2) translocation, and (3) desorption (Hamilton, 1998; Chmurzyńska, 2006). FAs are first delivered to the cell surface where they intercalate between outer leaflet phospholipids (adsorption). Next, a transition between outer and inner leaflet occurs (translocation) followed by exit from the plasma membrane into the cytoplasm (desorption). Acylation concomitant to desorption, termed vectorial acylation, may serve as a final step in FA transport and a means of directing the FA toward its target organelle (Zou et al., 2003). The integral membrane protein and putative lipid transporter CD36 along with fatty acid transport proteins (FATPs) have been suggested to carry out the first two steps and act as a docking site for plasma membrane associated fatty acid binding protein (FABP_pm_), which has been proposed to facilitate the desorption step. However, FABP_pm_ has also been proposed to function as a buffering system, maintaining the concentration of unbound fatty acids across the plasma membrane (Figure 2) (Abumrad et al., 1999; Glatz and Luiken, 2020). Cytoplasmic FABPs shuttle FAs to various organelles including the mitochondria for β-oxidation, the ER for lipid synthesis, or the nucleus where FAs regulate transcription.

### Fatty Acid Transport Proteins (FATPs)

The FATP family is considered the primary transporter in the putative protein-mediated FA transport mechanism. There are six isoforms in mammals, and each displays a unique pattern of tissue expression. They are members of the solute carrier protein superfamily, classified specifically as Slc27a1-6. FATP1 was the first family member discovered in 1994 and is most highly expressed in skeletal muscle and adipose tissue. FATP1 was shown to localize to the plasma membrane and significantly increased radiolabeled oleic acid uptake when overexpressed in the adipocyte 3T3-L1 cell line (Schaffer and Lodish, 1994). The structure of FATPs is unknown but based on sequence and protease protection assays of epitope tagged FATP1, FATPs are oriented with an extracellular N-terminus and a cytoplasmic C-terminus (Lewis et al., 2001; Stahl, 2004). FATPs contain one or more membrane-spanning regions, multiple membrane-associated regions, and an AMP-binding motif in the intracellular region. FA transport through FATP is ATP-dependent, and the sequence of the AMP-binding region is required for transport. Loss-of-function mutations in the AMP-binding domain prevent FA uptake, suggesting AMP-binding is directly involved in or coupled to the transport mechanism (Stuhlsatz-Krouper et al., 1998). Further, overexpression of FATP1 increased intracellular FA acylation, thus suggesting a role for FATPs in cellular acyl-coA synthetase activity (Steinberg et al., 1999). These results, along with sequence similarity to acyl-CoA synthetases, suggested that FATPs function to transport FAs through the plasma membrane and esterify them as they enter the cell. Evidence has emerged suggesting some FATPs do not actually transport FAs, but rather enhance FA uptake through esterification at the ER, thus lowering intracellular levels of non-esterified FAs and encouraging FA uptake (Milger et al., 2006). Whether the transport and esterification functions of FATP are coupled or independent is widely debated and may depend on the cellular context.

FATP1 is the primary member expressed in adipocytes. Translocation to the plasma membrane is stimulated by insulin and causes an increase in FA uptake (Kim et al., 2004). This insulin stimulation is consistent with the current model that inhibition of lipolysis through the intracellular hormone sensitive lipase (HSL) signals a shift toward glucose utilization and storage of TGs. FATP1 KO mice showed delayed clearance of serum FAs following insulin injection. FA uptake in primary brown adipocytes from these mice was diminished following insulin treatment, suggesting FATP1 is needed to transport FA from the circulation into tissue. Radiolabeled oleic acid uptake into WAT in FATP1 KO mice was diminished, while uptake by the liver and heart was increased, demonstrating a systemic compensation for disruption of FA transport by specific FATPs (Wu et al., 2006). Single nucleotide polymorphisms (SNPs) in FATP1 are linked to increased plasma TG levels in humans, and high expression of other FATPs in peripheral tissue correlates with obesity and insulin resistance owing to an accumulation of intracellular FAs (Lobo et al., 2007).

FATP1 in BAT is necessary for maintaining core body temperature after prolonged cold exposure (Wu et al., 2006). It is primarily localized to the plasma membrane upon cold activation, but a small pool is detected on intracellular membranes as well. Besides uptake of exogenous FAs, FATP1 may aid in lipid transport between organelles in brown adipocytes or esterify incoming FAs for quick turnover into β-oxidation. FATP1 expression is significantly induced in cold-activated BAT, and FATP1 KO mice exhibit a significant drop in body temperature after ~12 h of cold exposure. This was accompanied by a rise in serum FA levels during cold exposure, likely owing to the inability of activated BAT to efficiently take up exogenous FAs. Peroxisome proliferator-activated receptor alpha (PPAR-α) and gamma (PPAR-γ), two transcriptional regulators of cell differentiation and lipid metabolism, were shown to control expression of FATP1 in 3T3-L1 adipocytes (Frohnert et al., 1999). Treatment with activators of PPAR-γ, such as linoleic acid, induced expression of FATP1 and FA uptake. This is consistent with the induction of both PPAR-γ and FATP1 in BAT during cold exposure, although a direct path of regulation has not been demonstrated in cold-activated BAT. Similarly, BAT-specific KO of FATP1 has yet to be explored to elucidate the function of its FA transport role in thermogenesis. Future studies are required to contextualize the role of FATPs in exogenous FA uptake by BAT.

### Fatty Acid Binding Proteins (FABPs)

FABPs are a family of intracellular protein chaperones that bind long chain fatty acids (LCFAs) to expedite movement through membranes. There are currently 9 FABPs named for the tissue of discovery, but they are not exclusively expressed in the tissue for which they are named. The cytoplasmic FABP family differs in sequence, structure, and function from the plasma membrane associated FABP (FABP_PM_). First described in 1985, the putative fatty acid transporter FAPB_PM_ was shown to have a high affinity for fatty acids (Stremmel et al., 1985). Later, FABP_PM_ was found to be identical to mitochondrial aspartate aminotransferase, a member of the malate-aspartate shuttle, although expression at the plasma membrane was associated with fatty acid uptake (Berk et al., 1990; Bradbury and Berk, 2000). Inhibition of FABP_PM_ by antibody treatment in multiple tissues yielded a decrease in LCFA uptake while overexpression in skeletal muscle yielded an increase, thus supporting FABP_PM_ as a lipid transporter (Berk et al., 1990; Clarke et al., 2004). More work is needed to understand the importance of FABP_PM_ expression in brown adipocytes and how it changes with cold exposure. The remainder of this section will focus on other FABP family members in the cytoplasm.

Cytoplasmic FABPs solubilize hydrophobic molecules in the aqueous cellular environment, acting as chaperones and delivery systems for lipids between organelles. Structural characterization has revealed an indirect role in LCFA uptake; that is, FABPs do not facilitate transport across the plasma membrane but are essential for delivery into the cell (Storch and McDermott, 2009). All FABPs share a common tertiary structure comprised of antiparallel beta sheets forming a small beta barrel within which a hydrophobic molecule may bind (Sacchettini et al., 1988). There are subtle structural differences between isoforms that dictate ligand specificity. For example, adipocyte FABP (FABP4), also known as adipocyte protein 2 (AP2), exclusively binds LCFA while liver FABP (FABP1) can additionally bind eicosanoids, lysophospholipids, and acyl-CoA (Rolf et al., 1995; Thompson et al., 1997; Furuhashi and Hotamisligil, 2008). These cytoplasmic FABPs have also been shown to be secreted into the bloodstream with several physiological stresses including high fat diet, insulin resistance, and cold exposure (Hotamisligil and Bernlohr, 2015; Shu et al., 2017).

The adipocyte associated FABP (FABP4) is the best characterized in its family. FABP4 was first isolated from mouse embryonic fibroblast (3T3)-derived adipocytes, although it is highly expressed in macrophages as well (Hunt et al., 1986; Hotamisligil et al., 1996). FABP4 KO mice were viable, developed normally, and were indistinguishable from control mice in appearance and metabolic health (Hotamisligil et al., 1996). Like control mice, they were sensitive to dietary and genetic obesity, but were protected from insulin resistance and diabetes. This implicates FABP4 in suppression of insulin signaling, perhaps through increasing the intracellular lipid pool. Additionally, these mice exhibited a compensatory increase in epidermal FABP (E-FABP or FABP5) expression in adipose tissue, despite basal FABP5 expression being almost 100-fold lower than FABP4. Besides binding FAs, FABP4 has been shown to suppress PPAR-γ, a master adipocyte transcription factor that activates genes involved in lipid synthesis and uptake to enhance adipogenesis (Garin-Shkolnik et al., 2014). FABP4 also stimulates intracellular lipolysis through direct binding of HSL as suggested by a reduction in lipolysis in FABP4 KO mice and demonstrated by fluorescence resonance energy transfer of FABP4 mutagenized within the putative HSL binding site (Smith et al., 2008). At a chemical level, FABP4 binds FAs entering the cell. However, substantial evidence illustrates its function as an intracellular signal for proper adipocyte function and systemic fuel utilization.

FABP4 is increased with cold exposure in BAT and in the blood plasma and was shown to be necessary for FFA uptake into BAT (Shu et al., 2017). Knockout of FABP4 and HFD led to cold sensitivity and lower UCP1 expression that could be rescued with recombinant FABP4. FABP4 was shown to be sufficient to drive increased oxygen consumption in mice and elevated UCP1 expression. This increase required FA binding to FABP4, as a mutant R126Q did not have the same rescue capacity. Mechanistically, FABP4 in the BAT was shown to drive the intracellular conversion of T4 to T3 which is necessary for the thermogenic program (Ahmadian et al., 2011). FABP4 has also been shown to be increased in the BAT of hibernating mammals (Hittel and Storey, 2001; Eddy and Storey, 2004). Other studies have shown that FABP4 and 5 are necessary for proper metabolic adaptation in cold-activated BAT and that fasting greatly impacts this contribution to BAT function as well. When fasted for 20 h prior to a 4-h cold exposure, FABP4/5 KO mice were cold intolerant but indistinguishable from control mice in a fed state (Syamsunarno et al., 2014). This suggests a requirement of FABP4/5 for proper adjustment of systemic fuel utilization during physiological stress, such as long-term fasting and cold.

Curiously, heart-type FABP (FABP3) has been shown to be the dominant isoform in BAT upon cold activation. Expression of FABP3 was significantly increased in BAT after 4 h of cold despite a basal level of expression 6-fold lower than FABP4. Further, FABP3 KO mice were cold intolerant but displayed similar changes in thermogenic transcriptional programs to WT mice, such as induction of the coactivator PGC1α, which regulates mitochondrial biogenesis. Expected physiological changes were observed in FABP3 KO mice as well, such as depletion of TGs in BAT and increased FFAs in plasma (Vergnes et al., 2011). FABP3 was also shown to increase in hibernating ground squirrels (Hittel and Storey, 2001). Together, these data highlight an essential role for FABP3 in the uptake of exogenous lipids into BAT, the dysfunction of which results in an inability to maintain body temperature. Although FABP3 is not the dominant isoform in BAT, its function in cold exposure suggests a dynamic response network of FABPs in certain cell types depending on the environmental stimulus (Daikoku et al., 1997; Yamashita et al., 2008). This also underscores the gaps in current knowledge of FABPs in BAT function.

### Cluster of Differentiation 36 (CD36)

CD36, also referred to as fatty acid translocase (FAT), is an integral membrane protein belonging to the scavenger receptor superfamily. SRs bind a host of ligands including LDL, oxidized LDL, phospholipids, cholesterol esters, collagen, thrombospondin, carbohydrates, and microbial pathogens. There are 11 classes of scavenger receptors categorized based on primary sequence (A-L). CD36 belongs to the scavenger receptor B class (SR-B), the members of which contain two transmembrane domains and a single extracellular loop (Pepino et al., 2014). Diversity in the extracellular loop allows SR-Bs to regulate a variety of signal transduction pathways. Loss of function mutations or genetic deficiencies of SR-B members causes dysregulation of apoptosis, inflammatory response, and intracellular metabolism resulting in metabolic disease (Bonen et al., 2007; Glatz et al., 2010; McFarlan et al., 2012).

Although originally identified in human platelets, CD36 was characterized as a high affinity long-chain fatty acid (LCFA) receptor and transporter in rat adipocytes (Abumrad et al., 1993; Harmon and Abumrad, 1993). Treatment with highly charged FA analogs (N-sulfosuccinimidyl LCFA esters) inhibited oleate transport into adipocytes, and covalent protein labeling identified the 88 kDa CD36 as the receptor candidate (Harmon and Abumrad, 1993). Northern blotting revealed transcript abundance across tissues including adipose, heart, intestine, and skeletal muscle. Oleate uptake was limited to cell lines expressing CD36, further implicating the membrane protein as a lipid transporter (Abumrad et al., 1993). Modern proteomics and transport kinetics studies in transgenic Chinese hamster ovary cells identified a definitive FA binding pocket in CD36 that supports a direct function in lipid binding (Kuda et al., 2013). This pocket contains lysine 164 which binds the CD36 inhibitor N-hydroxysuccinimidyl ester of oleate (SSO) and, when mutagenized to alanine, diminished FA uptake. Despite a host of evidence supporting its lipid binding function, its role as a transporter has long been disputed.

Transmembrane LCFA transport has been at the center of a contentious debate for decades. The two opposing sides are comprised of “diffusionists,” who argue for rapid diffusion-mediated LCFA transport, and “translocationists,” who suggest transport is facilitated by proteins (Pownall and Moore, 2014; Glatz and Luiken, 2020). There is a myriad of evidence supporting both sides. The diffusionists argue that in biological membranes, lipids constantly diffuse laterally and often exchange between membrane leaflets without proteins. The FFAs would transfer from albumin to the outer leaflet of the plasma membrane and then flip-flop to the inner leaflet (Hamilton, 1998). This hypothesis is supported by demonstration of diffusion through fluorescent LCFA uptake in lipid vesicles pre-treated with inhibitors of FA transporters, two of which were direct competitive inhibitors for CD36 (Jay et al., 2020). The rate of uptake in rat adipocytes was unaffected by inhibitor treatment, suggesting that proteins are not required for transport (Abumrad et al., 1993; Harmon and Abumrad, 1993; Coburn et al., 2000; Kuda et al., 2013). However, these are imperfect systems to study lipid transport. Artificial membranes do not reflect true biological lipid or protein composition, and cultured cells do not reflect membrane dynamics. For example, fatty acid chain composition is different in culture, having lower levels of poly-unsaturated fatty acids compared to cells in organisms (Else, 2020).

The translocationists have been supported by several key findings including the observation that FFA uptake can be saturated in multiple cell types at levels within physiological range and that the flip-flop between the outer and inner leaflet does not occur at high enough levels to support metabolic demand (Kleinfeld and Storch, 1993; Kleinfeld et al., 1997). Moreover, an antibody to CD36 blocked FFA uptake into mouse adipocytes, and mutation of Lys164 in the FA binding pocket demonstrates site specific functional regulation (Abumrad et al., 1993; Pepino et al., 2014). Recent work has found that palmitoylation of CD36 leads to caveolae-dependent internalization, and inhibition of palmitoylation prevents this uptake (Hao et al., 2020). While the mechanism of lipid transport remains controversial, the *in vivo* role of CD36 in regulating FA uptake and metabolism has been demonstrated across many models. Moreover, in humans, common SNPs in the promotor of *CD36* cause protein deficiency and have been associated with high levels of FAs and LDL in serum, a phenotype recapitulated in *Cd36*^−/−^ mice (Miyaoka et al., 2001; Ma et al., 2004; Goudriaan et al., 2005; Yamashita et al., 2007; Love-Gregory et al., 2011). Newer models favor both diffusion and translocation that are important in different ranges and energy demands of the cell (Abumrad et al., 1999).

CD36 expression is known to be highest in adipose tissue, and it is required for body temperature regulation in mice (Putri et al., 2015). Additionally, its expression is significantly increased in BAT following cold exposure (Bartelt et al., 2011). CD36 is required for lipoprotein uptake in BAT, and global CD36 KO in mice results in TG buildup in BAT and cold intolerance (Yamashita et al., 2007; Bartelt et al., 2011). CD36 is necessary for transport of lipids via lipoproteins and FFA-bound albumin in BAT as well. Clearance of both lipoproteins and FFAs from plasma and uptake into BAT were diminished in cold-exposed Cd36 KO mice (Bartelt et al., 2011). CD36 is also required for uptake of lipid-related molecules into BAT, such as coenzyme Q (CoQ) (Anderson et al., 2015). CoQ contains an isoprenoid tail and is a necessary electron transporter between complexes in the electron transport chain. Therefore, it is required for both ATP synthesis and heat production in BAT. High levels of CoQ are present in BAT despite low levels of endogenous synthesis, suggesting a requirement for uptake of CoQ from the circulation. Whole-body *Cd36*^−/−^mice displayed a 2-fold increase in serum CoQ accompanied by CoQ deficiency, TG accumulation, and diminished mitochondrial size and metabolic capacity in BAT. Although CD36 is present in other tissues, these phenotypes were not reflected throughout the mice. Cold intolerance in both whole-body and BAT-specific *Cd36*^−/−^was driven by CoQ insufficiency causing reduced FA β-oxidation in BAT. These studies highlight the requirement of CD36 for proper uptake of lipids and hydrophobic molecules into cold-activated BAT.

## Extracellular Lipolysis and Ldl Receptor Endocytosis Facilitates Triglyceride and Cholesterol Uptake Into Bat

### Lipoprotein Metabolism and Regulation by BAT

Lipoproteins are means of transporting lipids of varying polarity from centers of lipid processing, such as the intestine and liver, to peripheral tissues through the circulation. They are comprised of a phospholipid monolayer interlaced with apolipoproteins that serve as structural reinforcements and as LDL receptor (LDLR) recognition sites to aid in endocytosis. Lipoproteins fall into four main classes depending on their relative composition of proteins and lipids: high density (HDL), low-density (LDL), very low-density (VLDL), and chylomicrons. In addition to the characteristic structure of each class, different lipoproteins also serve different functions. For example, LDL carries circulatory cholesterol and easily enters arterial walls, while chylomicrons are essential for dietary lipid transport to the liver and peripheral tissues. Nonetheless, each class has a vital role in lipid transport, endocytosis, and hydrolysis (Zanoni et al., 2018).

**Figure 3:**
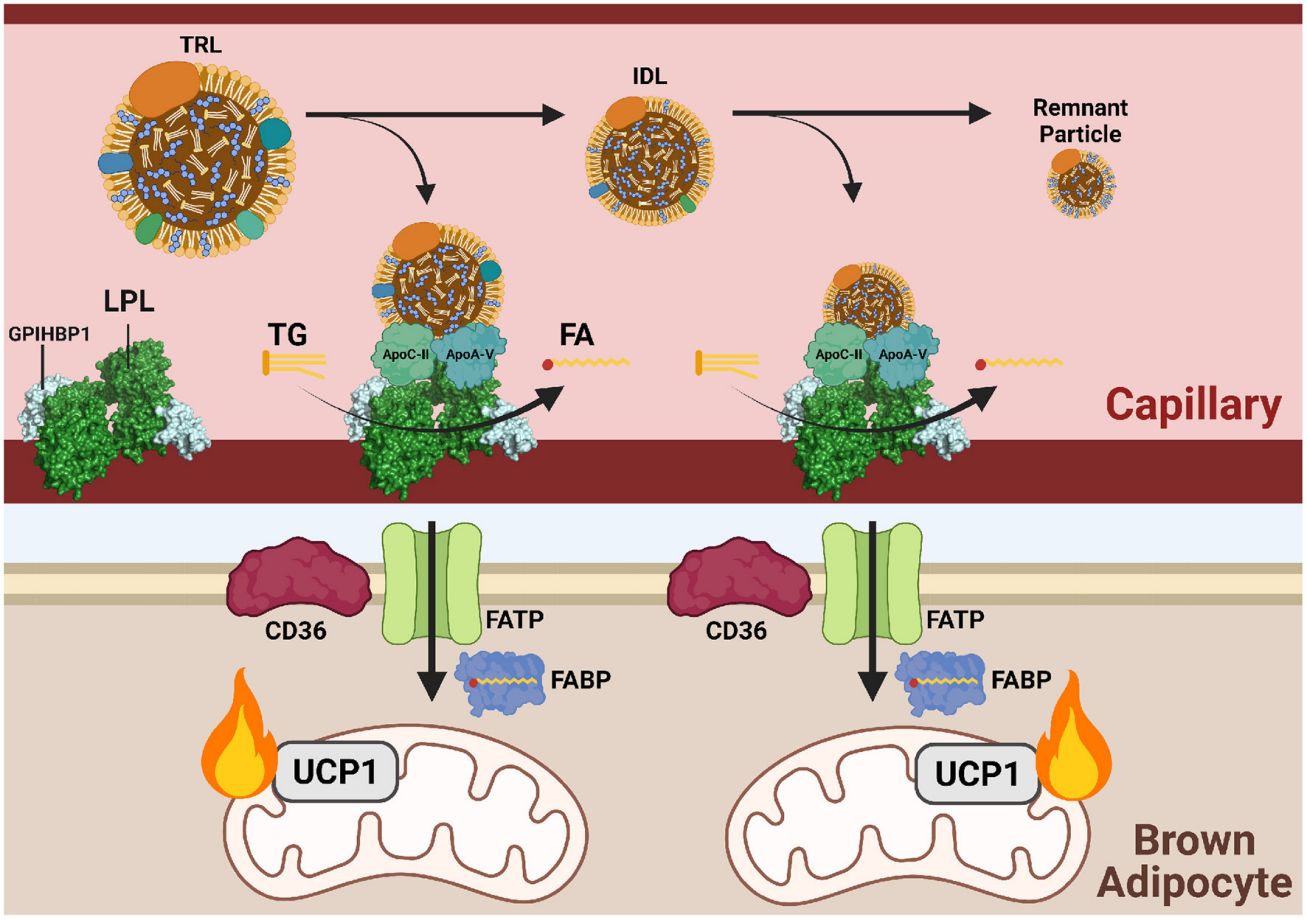
Triglycerides are delivered to cold-activated BAT through LPL hydrolysis at the endothelial wall. Following β3-adrenergic receptor (B_3_AR) activation by norepinephrine (NE) signaling a drop in temperature, hepatocytes and enterocytes mobilize triglyceride (TG)-rich lipoproteins (TRLs) into the blood for transport to brown adipocytes. Lipoprotein lipase (LPL) at the vascular endothelium hydrolyzes TGs from TRLs into fatty acids (FAs) for subsequent FATP/CD36-mediated transport into brown adipose tissue (BAT). Hydrolysis is dependent on the co-factor apolipoprotein C-II (apoC-II) and activator apolipoprotein A-V (apoA-V). As hydrolysis occurs, TRLs reduce into intermediate lipoproteins (IDLs), low-density lipoproteins (LDL), and finally remnant particles to be cleared by the liver. Each reduction in size is accompanied by a loss of triglycerides (TGs). Imported FAs are funneled into mitochondrial β-oxidation and eventual heat production.

LDL endocytosis begins with recognition at the cell membrane by a low-density lipoprotein receptor (LDLR). Following recognition, a clathrin-coated pit forms a vesicle around the endocytosed lipoprotein. The vesicle is directed to endosomes where a drop in pH causes release of LDL from LDLR. Acidic lipases in the newly formed lysosome hydrolyze the contents of LDL to release FAs from TGs and unesterified cholesterol from cholesteryl esters (Figure 3). Cholesterol is incorporated into cell membranes and negatively regulates its own synthesis by preventing translocation of sterol regulatory binding protein (SREBP) from the ER to the Golgi, thereby blocking transcriptional activation of HMG-CoA reductase (Brown and Goldstein, 1997). FAs released from LDL are shuttled to the ER for synthesis into membrane lipids, the Golgi for protein acylation, and the mitochondria for FA β-oxidation.

**Figure 4:**
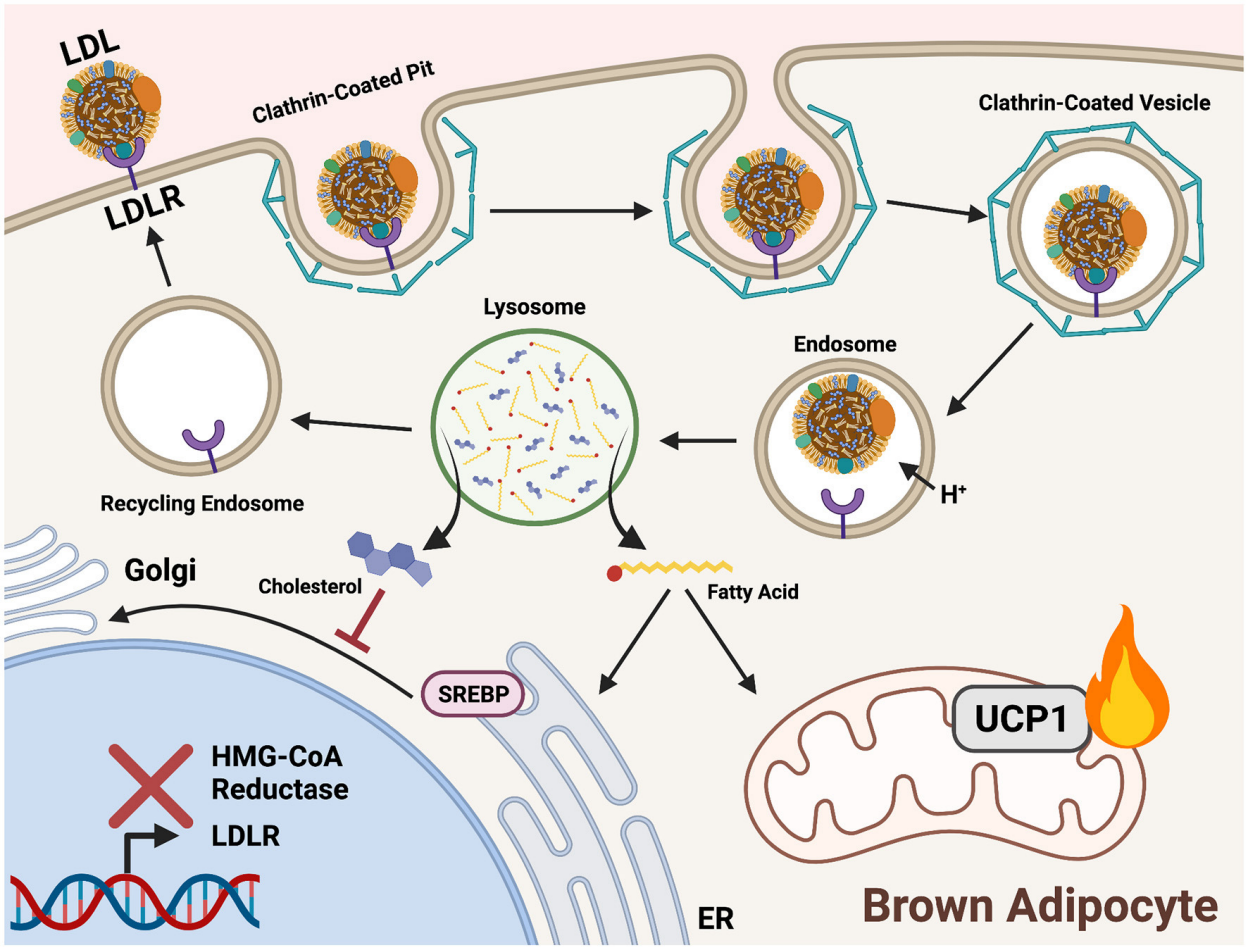
Triglycerides and cholesterol are delivered to cold-activated BAT through LDLR mediated endocytosis. Low-density lipoprotein receptors (LDLRs) recognize apolipoprotein B on the surface of low-density lipoproteins (LDL). A clathrin-coated pit forms around the bound receptor and an endocytotic vesicle forms. An internal drop in pH prompts release of LDL from LDLR for hydrolysis in the lysosome. Acid hydrolases liberate fatty acids (FAs) from triglycerides (TGs) and cholesterol within LDL. In brown adipocytes, FAs are shuttled into mitochondrial β-oxidation to fuel downstream heat production. Cholesterol is incorporated into cell membranes and signals a negative feedback loop of biosynthesis by preventing sterol-regulatory-element binding proteins (SREBP) translocation to the Golgi from the ER. This prevents proteolytic processing and localization to the nucleus, thereby inhibiting expression of genes encoding cholesterol synthesis enzymes (HMG-CoA reductase) and lipoprotein receptors (LDLR).

In brown adipocytes, LDLR endocytosis fuels the electron pool needed for proton gradient uncoupling and heat production (Figure 4). LDLR is recycled back to the plasma membrane for addition rounds of endocytosis (Ikonen, 2008). LDLRs are also necessary in systemic cholesterol and triglyceride balance during cold exposure. *Ldlr*^−/−^ mice displayed a reduction in plasma TG upon activation of BAT by cold or with a β3 adrenergic receptor agonist but did not show a reduction in plasma cholesterol normally observed after BAT activation (Dong et al., 2013; Berbée et al., 2015). In this model, the liver is unable to clear lipoprotein remnants produced from FA uptake in BAT following LPL-mediated TG hydrolysis and thus cholesterol remains in the circulation. Coordinated plasma lipid clearance between the liver and BAT during cold exposure requires proper protein-mediated lipid uptake, such as LDLR endocytosis.

### Triglyceride Uptake Into BAT

TGs are the neutral storage form of FAs and travel between tissues through the bloodstream via chylomicrons and VLDL. Chylomicrons are the largest form of lipoprotein and are assembled by the small intestine following emulsification of dietary fats. TGs packaged into chylomicrons mainly enter adipose tissue and skeletal muscle due to high lipoprotein lipase (LPL) activity. The remaining TG is taken up by the liver and can be repackaged into VLDL for use throughout the body. Alternatively, FAs are synthesized by the liver through *de novo* lipogenesis, assembled into TG, and mobilized in VLDL. As TGs are liberated from VLDL for use by peripheral tissues, smaller remnants of decreasing TG content are formed such as intermediate-density lipoproteins (IDL) and LDL. Like chylomicrons, VLDL is utilized by tissues after lipolysis by LPL.

LPL is the predominant lipase in tissues with high levels of exogenous lipid uptake, such as adipose tissue, heart, and skeletal muscle. LPL is a dimeric enzyme localized to the vascular lumen where it hydrolyzes plasma TGs into glycerol and FAs for tissue uptake. TG-derived FA uptake into BAT is reliant on localized LPL activity, as injection of an LPL inhibitor (tetrahydrolipstatin) or treatment with heparin to release LPL from the vascular wall in mice prior to cold exposure almost completely abolished labeled TRL uptake into BAT.^20^ LPL is ferried and anchored to the endothelium via glycophosphatidylinositol-anchored HDL-binding protein (GPIHBP1). Mice lacking GPIHBP1 have increased plasma TGs due to reduced tissue uptake (Cushing et al., 2018). As circulating lipoprotein levels are highly modulated in response to metabolic state, such as fasting and cold exposure, tight regulation of LPL activity is required. Besides localization, LPL is primarily regulated post-translationally by several extracellular proteins. Angiopoietin-like proteins (ANGPTL) are the main class of LPL regulators. ANGPTLs are secreted into the lumen and directly interact with LPL, preventing its dimerization and inhibiting lipolysis (Hegele, 2016).

ANGPTL4 is the predominant isoform in brown adipose tissue. In BAT, ANGPTL4 expression is induced during periods of nutrient deprivation (fasting) and suppressed when substantial nutrients are available or needed, such as postprandially or during cold exposure (Singh et al., 2018). In cold-activated WAT, ANGPTL4 is upregulated to shift away from fat storage and toward output of FAs to support thermogenesis. BAT-specific ANGPTL4 KO mice exhibit reduced plasma TGs in the fed and fasted state, accumulation of ^3^H from labeled triolein in BAT, and increased expression of CD36 in BAT (Cushing et al., 2018). LPL expression was unchanged, but activity was significantly increased in BAT from ANGPTL4 KO mice. ANGPTL4 KO mice had higher rectal temperature over time during a 4-h cold exposure, likely due to enhanced LPL activity in BAT fueling FA uptake and β-oxidation. In contrast, *Gpihbp1*^−/−^ mice show no change in labeled triolein uptake into BAT. Double GPIHBP1/ANGPTL4 KO mice show a partial correction in plasma TG levels compared to GPIHBP1 KO mice (Dijk et al., 2015). However, this correction is lost after a 2-week HFD, with both GPIHBP1 KO and GPIHBP1/ANGPTL4 KO showing a similar increase in plasma TGs compared to mice of the same genotype on a normal chow diet. In sum, these studies highlight the necessity of tight LPL regulation in BAT to modulate TG and FA uptake during metabolic stress, including cold exposure. It has been shown that BAT relies more heavily on LPL-based uptake of TG-derived FAs rather than particle endocytosis, but cold exposure significantly enhances the uptake of TGs through both methods (Khedoe et al., 2015). This was observed through tracing of ^3^H-triolein and ^14^C cholesteryl esters which allow measurement of TG uptake by lipolysis and lipoprotein uptake by endocytosis, respectively.

While VLDL is overwhelmingly shuttled to BAT when activated by exposure to cold, transport of HDL to the liver is drastically increased (Schaltenberg et al., 2021). HDL balances cholesterol flux between peripheral tissues and is both synthesized and excreted by the liver. This hepatic processing is dependent on endothelial lipase (EL). Following extended cold exposure (1 week), expression of the gene encoding EL, *Lipg*, was increased in murine BAT. Cold exposure was also shown to enhance HDL clearance from plasma. Moreover, *Lipg*^−/−^ mice displayed higher plasma levels of HDL, indicating poor cholesterol clearance. The HDL particles from *Lipg*^−/−^ mice were enriched in phospholipids but lacking in cholesterol and TG compared to WT mice both at room temperature and after prolonged cold exposure. EL is known to promote TRL uptake, much like LPL. While EL expression is induced in BAT after cold exposure, it was not required for proper thermogenic activation, nor did its loss affect thermogenic transcriptional programs such as those controlled by PPAR-γ.

## Various Lipids Altered in the Plasma With Cold Exposure

The advent and expansion of mass spectrometry based lipidomics has broadened the field of circulating lipids, and several lipid classes have been shown to be increased in blood plasma with cold exposure including acylcarnitines, ceramides, 12,13-dihydroxy-9z-octadecenoic acid (12,13-diHOME), and fatty acid esters of hydroxy fatty acids (FAHFAs). Little is known about how theses lipids are transported across plasma membranes, what functional roles they serve in non-shivering thermogenesis, and in what complex structure they are mobilized in the circulation. While this remains an emerging field, it is rapidly progressing with new mass spectrometry-based technology such as ion mobility, matrix-assisted laser desorption/ionization (MALDI) imaging mass spectrometry, heavy isotope labeling, and several new chemical probes (Kyle et al., 2018; Kirkwood et al., 2021).

### Acylcarnitines

Acylcarnitines are fatty acids conjugated to a carnitine through esterification. At the cellular level, acylcarnitines function as intermediaries facilitating transport of FFAs into the mitochondria for β-oxidation. Carnitine palmitoyltransferase 1 (CPT1) is embedded on the outer-surface of the mitochondrial membrane and esterifies the fatty acid from an acyl-CoA to carnitine. This acylcarnitine can then diffuse into the porous outer mitochondrial member. Acylcarnitine is then brought into the inner mitochondria by carnitine acylcarnitine transferase (CACT) and de-carnitylated by CPT2. Besides their cellular role for fatty acid transport, acylcarnitines are also found in the blood plasma. Plasma acylcarnitines increase with chronic diseases such as type 2 diabetes, cardiovascular disease, and inborn errors of metabolism as well as in acute metabolic stresses such as fasting, exercise, and cold exposure (Muoio et al., 2012; Schooneman et al., 2013; McCoin et al., 2015; Simcox et al., 2017). The functional role of acylcarnitines in the plasma has been proposed to range from protection from toxicity to a distinct storage pool that can be pulled from during energy demanding conditions (Muoio et al., 2012).

In cold exposure, short chain, medium chain, and long chain acylcarnitines are increased in the plasma while carnitine levels decrease (Simcox et al., 2017; Pernes et al., 2021). This cold induction of increased plasma acylcarnitines is mediated by β3-adrenergic receptor induced WAT lipolysis, since adipose tissue-specific ATGL knockout mice had no changes in acylcarnitine levels with β3-adrenergic receptor agonist treatment. Once FFAs are released from the WAT, they are taken up into the liver, where they transcriptionally activate CPT1, CACT, and CPT2 through an HNF4α-mediated mechanism as well as serve as substrate for acylcarnitine production (Simcox et al., 2017; Jain et al., 2021). These liver-produced acylcarnitines are then taken up into the BAT, skeletal muscle, and heart. In the BAT, the acylcarnitines are catabolized as a fuel source for thermogenesis. Beyond the liver, there are other potential sources for cold induced plasma acylcarnitines; while ablation of acylcarnitine production in the liver causes cold intolerance, it is not sufficient to completely block the rise in plasma acylcarnitines with cold exposure. Recently it has been shown that the kidney may also contribute to the plasma acylcarnitine pool (Jain et al., 2021). These studies collectively demonstrate that plasma acylcarnitines are produced through a multi-tissue processing, and that they function as a fuel source for cold-activated BAT.

Several questions remain in understanding the regulation and transport of plasma acylcarnitines including how they are transported through the plasma membrane in the liver and in the brown adipose tissue. Studies in Xenopus oocytes have demonstrated that acylcarnitines require a transporter to cross the plasma membrane, and cDNA libraries from mouse liver have demonstrated that these unknown transporters are present in the liver (Berardi et al., 1998; Nakanishi et al., 2001). SLC22a1 was recently identified as an acylcarnitine exporter in the liver, and knockout of SLC22a1 led to decreased short and medium chain acylcarnitines in plasma but had no impact on long chain acylcarnitine levels (Kim et al., 2017). Moreover, there has been no identified BAT acylcarnitine transporter and SLC22a1 has low expression in brown adipocytes. Plasma long chain acylcarnitines have been shown to travel bound to albumin, while short and medium chain acylcarnitines are unbound. Whether albumin bound acylcarnitines are the dominant form of acylcarnitine in the circulation during cold exposure is unknown. Future work will be needed to understand their entry into brown adipocytes and the kinetics of their uptake compared to FFAs of the same acyl chain.

### Ceramides

Ceramides are a long chain sphingoid base conjugated to a fatty acid through an amide bond and are the precursor to all sphingolipids. Ceramides are known to circulate during tissue dysfunction and metabolic disease in both mice and humans. Plasma ceramide levels have been shown to correlate with risk of diabetes and coronary artery disease in a species-specific manner across human cohorts (Tippetts et al., 2021). Reduction of plasma and WAT ceramides in mice via increased degradation (ceramidase overexpression) or inhibition of ceramide synthesis (SPTLC2 KO) ameliorated HFD-induced obesity, insulin resistance, and hepatic steatosis (Xia et al., 2015; Chaurasia et al., 2016). SPTLC2 KO in WAT also enhanced adipocyte browning and resulted in an increase in beige adipocyte differentiation (Chaurasia et al., 2016). This suggests that ceramides act as signals to increase lipid storage in WAT and inhibit the beige program. Moreover, liver SPTLC2 expression is upregulated in response to SPTLC2 KO in WAT, suggesting a means of communication to balance tissue ceramide levels.

Despite the wealth of literature on ceramide function in metabolic disease and its regulation of the adipocyte differentiation program, little is known about how ceramides control brown and beige adipocyte maintenance or their direct role in thermogenic metabolism. We have observed significant increases in plasma ceramides following acute cold exposure, with computational assessment revealing that these plasma levels are regulated by the BAT and the kidney (Jain et al., 2021). More work is needed to characterize how these plasma ceramides are regulated in acute cold exposure, what their functional role may be, and what complexes facilitate their transport in the plasma during cold exposure. At ambient temperature, ceramides are known to be associated with lipoproteins (primarily LDL) and have been shown to transfer between cells via extracellular vesicles (EVs) (Hammad et al., 2010; Crewe et al., 2018). These vesicles act as carriers for intercellular signaling molecules during metabolic stress, and many ceramide species act as second messengers for key metabolic pathways including insulin sensing and cell growth. For example, during fasting, white adipose tissue traffics EVs containing signaling molecules such as caveolin 1 and very long chain ceramides to neighboring endothelial cells (and vice versa) (Crewe et al., 2018). EVs are also produced by adipose-derived stem cells during beige adipocyte differentiation and were shown to be sufficient to differentiate these stem cells into beige adipocytes (Jung et al., 2020). Additionally, BAT has been shown to be a significant contributor of exosomes into the circulation (Thomou et al., 2017). It is unknown whether export of these vesicles is upregulated or if ceramides are enriched in these vesicles during cold exposure. Moreover, there are no known plasma membrane transporters of ceramides. Ceramides in cold exposure remain an exciting area of research with many outstanding questions, including the role of ceramides in thermogenic metabolism as well as the function of plasma ceramides compared to ceramides produced in brown and beige adipocytes.

### 12, 13-Dihydroxy-9Z-Octadecenoic Acid (12,13-DiHome)

12,13-diHOME is produced in brown adipose tissue and can function as an autocrine or paracrine signal to increase mitochondrial oxidation rates (Lynes et al., 2017). Upon β3-adrenergic receptor activation, linoleic acid is oxidized by cytochrome P450 and soluble epoxide hydrolase to produce 12,13-diHOME. Beyond BAT, other tissues are known to produce 12,13-diHOME, including the skeletal muscle, and contribute to the circulating pool to regulate body weight, energy expenditure, insulin sensitivity, and plasma lipid levels (Vasan et al., 2019). The uptake of 12,13-diHOME into brown adipocytes is regulated by CD36 and FATP1, and treatment of brown adipocytes with 12,13-diHOME is sufficient to increase translocation of CD36 and FATP1 (Lynes et al., 2017, 2019). Further studies are needed to understand how 12,13-diHOME regulates mitochondrial oxidation, determine the mechanism of secretion from cells, and understand how this lipid circulates in the plasma.

### Fatty Acid Esters of Hydroxy Fatty Acids (FAHFAs)

FAHFAs are a recently discovered class of signaling lipids that regulate brown and beige adipocyte differentiation and maintenance. Structurally, FAHFAs are fatty acids complexed to a hydroxy fatty acid through an ester bond (Yore et al., 2014). There are numerous types of FAHFAs named for the acyl chains and the location of the hydroxylation including stearic-acid-9-hydroxy stearic acid (9-SAHSA), oleic-acid-9-hydroxy stearic acid (9-OAHSA), and palmitic-acid-9-hydroxy stearic acids (9-PAHSA). Both 5- and 9- PAHSA have been shown to increase brown adipocyte differentiation, insulin sensitivity, decrease inflammation in adipose tissue, and improve whole body glucose tolerance. Treatment of 3T3-L1 adipocytes or leptin deficient mouse models with 9-PAHSA led to increased expression of thermogenic genes including UCP1 (Wang et al., 2018). Part of this signaling is mediated through binding and activating G-protein coupled receptor 120 (GRP120), and knockdown of GPR120 in 3T3-L1 cells abrogated the effect of 9-PASHA treatment (Oh et al., 2014; Wang et al., 2018). Cold exposure induced the production of 5- and 9-PAHSA from WAT, with this production being mediated by lipolysis from triglycerides since knockout of ATGL led to ablated the cold-induced production (Paluchova et al., 2020). Many outstanding questions remain on how various species of FAHFAs impact brown and beige adipocytes and how they are transported into cells.

The numerous plasma lipids that act upon BAT is still an open area of study. For the purpose of this review, we have chosen to focus on plasma lipids that are transported into brown adipocytes, however there are a number of other lipids that are altered in brown adipocytes themselves that regulate thermogenesis. These include ether lipids (such as plasmalogens) and cardiolipins (Lynes et al., 2018; Park et al., 2019; Von Bank et al., 2021b). Although FFAs produced in the BAT are not necessary for thermogenesis (Schreiber et al., 2017), they have been shown to be sufficient to drive the thermogenic program through mediation of GPCR signaling (Sveidahl Johansen et al., 2021). Interestingly, ether lipids have recently been observed to increase with cold exposure in studies where mice were acclimated to thermoneutrality then placed for 24 h in thermoneutrality, room temperature (22°C), and cold exposure (5°C) as well as fasted for the final 5 h of temperature stress (Pernes et al., 2021). More work is needed to understand these various lipids in the plasma and how their transport is regulated.

## Perspectives

BAT is an important regulator of whole-body glucose and lipid homeostasis. Cold exposure increases the uptake of lipids into the BAT by 12-fold and, in models of hyperlipidemia, can normalize plasma triacyclglycerol and cholesterol levels (Bartelt et al., 2011). Not only are thermogenic adipocytes able to regulate systemic lipid metabolism, but they are also reliant on the plasma lipid pool for fuel availability. The importance of peripheral lipid storage for non-shivering thermogenesis has now been established through use of the ATGL KO studies and DGAT1 and 2 double KO studies (Schreiber et al., 2017; Chitraju et al., 2020). The uptake of these lipids into BAT from the circulation is dependent upon facilitated transport through dedicated protein transporters, chaperones, and endocytosis. This review focused on known mechanisms of lipid uptake into BAT, beginning with FFA uptake which is regulated in three distinct steps: CD36 and FATPs regulating (1) adsorption and (2) translocation, while FABP facilitates (3) desorption (Hamilton, 1998; Chmurzyńska, 2006). Loss of CD36, FATP or FABP led to cold intolerance and an inability for cold exposure to regulate circulating FFA levels. TGs and cholesterol can also be imported into BAT through LDL endocytosis, or for TGs, through LPL mediated lipolysis from TRL. While the majority of work has focused on uptake of FFAs, TGs, and cholesterol into BAT, questions remain on the import mechanisms that regulate other plasma lipids during cold exposure. Recent work on plasma acylcarnitines has shown that they are taken up by BAT and are necessary for thermogenic capacity (Simcox et al., 2017). Other work has shown that lipid containing exosomes are increased in the plasma with cold exposure and reflect brown adipocyte activity (Chen et al., 2016). More work is needed to understand how these lipids and lipid-containing vesicles are trafficked into cells, and to determine the tissues where these various plasma lipids are produced.

One existing challenge in the modeling of lipid uptake into brown adipocytes is standardization of protocols for cold exposure and mouse models. Many studies have a range in cold exposure from 3 h to 1 week. Longer cold exposure, such as 72 h to 1 week, is associated with beige adipocyte differentiation, increased BAT mass, and increased food intake (Ikeda et al., 2018). The variations in cold exposure timing and added variable of fasting during a traditional cold tolerance test make comparison difficult due to differences in thermogenic capacity and contribution of the beige depot. Moreover, variations in housing temperature also impact the brown and beige adipocyte population and alter body weight in response to shifts in energy expenditure (Fischer et al., 2018; Corrigan et al., 2020). Many of the studies associated with lipid uptake in brown adipocytes focus on 1 week including characterization of FABP and FATP. Standardization would enable an understanding of the impact of BAT on the circulating lipid pool.

Another barrier for lipid transport in thermogenic adipocytes is depot-specific gene modulation. Many mouse models for lipid transport assessed the function in BAT using ablation of the gene in all adipose tissue with cre expression driven by the adiponectin promoter or in whole body KO models. All of the work to assess FATP1 function in BAT was performed in FATP1 null mice, as were several of the seminal studies on CD36 (Lobo et al., 2007; Bartelt et al., 2011). Models that use cre drivers target both the brown and beige adipocytes using UCP1-cre for genetic modulation of various genes. An important step in furthering our understanding of lipid transport in thermogenesis will be the development of mouse models that target only the brown or beige adipocytes. Single cell sequencing has uncovered numerous unique markers of beige vs. brown adipocytes, while also identifying numerous sub-populations of adipocytes in brown and beige depots (Merrick et al., 2019; Henriques et al., 2020; Sun et al., 2020). These challenges are particularly important since the uptake of lipids into each of these cell types may be mediated by distinct membrane composition and expression of transporters.

Finally, although the majority of this review focused on lipids being transported into BAT for catabolism, lipids are capable of playing a number of signaling roles that regulate thermogenic potential. Recent work by the Seale group has demonstrated that FA oxidation is an important mediator of beige adipocyte differentiation driven by transcriptional regulator PRDM16. The breakdown of these FAs into ketone bodies was necessary and sufficient to induce differentiation of pre-adipocytes into beige adipocyte (Wang et al., 2019). Beige adipocyte differentiation was also shown to be regulated by ceramide signaling which inhibits the beige program while promoting lipid accumulation needed for white adipocytes (Chaurasia et al., 2016). More work is needed to understand how lipids influence metabolism in brown and beige adipocytes and how they contribute to the thermogenic potential, as well as how these signals are mediated by transport into the BAT.

Lipid import from the circulation into brown adipocytes is necessary for thermogenesis. Once in the brown and beige adipocytes, these lipids can be catabolized as an energy source or serve as signaling molecules. While there are fairly established mechanisms and function for FFA and TG uptake into BAT, more work is needed to characterize the uptake, circulating form, and functional role in thermogenesis for other lipids such as acylcarnitines, ceramides, and FAHFAs. The continued exploration and development of new technology to probe lipid uptake in brown and beige adipocytes will enable distinction of catabolism, storage, and signaling capabilities. Lipid transport proteins are essential to proper systemic lipid metabolism, and tight regulation of this transport is necessary to prevent disease.

## Author Contributions

GW, AM, and JS contributed to the conception, writing, literature searches, and first draft of the manuscript. GW created the figures for the manuscript. JN and JS contributed editing, revisions, literature searches, and final draft overview. All authors contributed to manuscript revisions, read, and approved the submitted version.

## Funding

Research reported in this publication was supported by the Eunice Kennedy Shriver National Institute of Child Health & Human Development of the National Institutes of Health, the Office of the Director, National Institutes of Health (OD) and the National Cancer Institute (NCI) under Award Number K12HD101368. The content is solely the responsibility of the authors and does not necessarily represent the official views of the National Institutes of Health. The work was also supported in part by startup funds from the University of Wisconsin-Madison School Department of Biochemistry to JS and NIH RO1 DK118093 to JN. Other funds that supported this publication include funds from the Diabetes Research Center at Washington University in St. Louis of the National Institutes of Health under award number P30DK020579.

## Conflict of Interest

The authors declare that the research was conducted in the absence of any commercial or financial relationships that could be construed as a potential conflict of interest.

## Publisher's Note

All claims expressed in this article are solely those of the authors and do not necessarily represent those of their affiliated organizations, or those of the publisher, the editors and the reviewers. Any product that may be evaluated in this article, or claim that may be made by its manufacturer, is not guaranteed or endorsed by the publisher.
